# Evaluating the predictive validity of four divergent thinking tasks for the originality of design product ideation

**DOI:** 10.1371/journal.pone.0265116

**Published:** 2022-03-14

**Authors:** Abbey K. Erwin, Khue Tran, Wilma Koutstaal

**Affiliations:** Department of Psychology, University of Minnesota, Minneapolis, Minnesota, United States of America; University of Oviedo, SPAIN

## Abstract

What factors predict the originality of domain-specific idea generation? Replicating and extending an earlier study using a Design Product Ideation task in an introductory university design course, the present research, grounded in the componential theory of creativity, assessed the relative contributions to originality of design ideation from five factors: divergent thinking, personality traits, general cognitive ability, prior creative experience, and task-specific challenge/interest. The Design Product Ideation task asked participants, at two different timepoints, to propose ideas for products to improve either the experience of urban gardening or of outdoor picnics. Four divergent thinking tasks were used, including the predominantly conceptually-based Alternative Uses Task, a newly developed perceptually-based Figural Interpretation Quest, and two modified verbal tasks from the Torrance Tests of Creative Thinking (Torrance Suppose and Torrance Product). Regression analyses revealed that, at both timepoints, originality on the Design Product Ideation tasks was predicted by multiple divergent thinking, personality, and task-based factors. Originality of responses to the Figural Interpretation Quest was a significant predictor at both timepoints, and continued to add incremental value after controlling for the other divergent thinking measures. Collectively, these findings indicate that the four divergent thinking tasks, though related, do not measure identical constructs, and that many individual difference components, both trait-based (e.g., openness to experience) and more specifically task-based (e.g., perceived challenge of the task), shape creative performance. Methodologically, and from a practical standpoint, these findings underscore the value of incorporating both conceptual and perceptual measures of divergent thinking as contributors to originality in domain-specific idea generation.

## Introduction

Creativity is a broad concept in psychology that involves developing new or alternative and valuable ideas to create art, solve problems, communicate, and more [1–3]. From within a componential model of creativity, creative performance is understood as being motivated by a number of personality, cognitive, and environmental factors [4–8]. Creativity is a key reason we are able to make progress in science and technology, as well as to create new music, movies and video games. Beyond these perhaps more commonly noted domains, creativity can also lead to solutions to social injustices and global inequity. Divergent thinking, a component of creative problem solving, is defined as a person’s ability to start with a singular object or idea and produce as many alternative solutions or representations as possible [9–11]. There are numerous measures available to evaluate divergent thinking, many serving as predictors of creative potential rather than directly measuring creative output [12].

Current research asks what factors influence creative performance, as well as interventions or training that can be used to improve creative performance [13–15]. Studies have focused on personality traits, previous experiences, attention, and executive functioning as individual difference factors related to creative performance [16–18]. Educational interventions, mindset prior to creative tasks, and the influence of specific instructions given to participants have all been explored as ways to improve creative performance [10, 16]. Divergent thinking tasks offer a valuable–but not frequently adopted–method to quantitatively assess training-related changes in creative thinking [19, 20]. In divergent thinking tasks, participants are asked to produce novel ideas about or interpretations of a given stimulus [9]. Divergent thinking tasks thus allow assessment of an individual’s generation of novel ideas under specific (controlled) task and stimulus conditions.

The present study evaluates the concurrent predictive validity of various measures of divergent thinking, personality, cognitive ability, previous creative experiences, and task-specific factors for a design task. The dependent variable in the study is performance on a Design Product Ideation (DPI) task that asks participants to produce product ideas or improvements based on one of two prompts: either a picnic or urban gardening. Originality scores are the primary focus of the study, as this measure indexes the number of novel ideas a participant produces that are relevant to the prompt, and originality is an especially strong correlate of creativity and innovativeness [1]. Our focus on how different domain-general divergent thinking measures contribute to Design Product Ideation complements recent efforts to assess the concurrent predictive value of different divergent thinking tasks to such domain-specific assessments as architectural design [21], creativity in the realm of advertising [22], and also earlier research that compared how different assessments of divergent thinking related to self-reported creativity [23]. As elaborated below, the current study also represents a conceptual replication and extension of our earlier research using the Design Product Ideation task [6] examining the concurrent predictive utility of four divergent thinking measures: the Alternative Uses Task, two modified verbal tasks from the Torrance Tests of Creative Thinking (Torrance Product and Torrance Suppose or Consequences) and the Figural Interpretation Quest.

The Alternative Uses Task (AUT) is a heavily researched measure of creativity designed to measure divergent thinking [9]. The task asks participants to name as many nontypical or unusual uses as possible for common objects such as a brick or paper clip, and so provides an assessment of the ability to produce diverging uses from a single starting point. Previous research has explored the cognitive mechanisms underlying the Alternative Uses Task using a think-aloud strategy to understand the strategies participants use to produce novel uses for everyday objects [24]. Results suggest that participants used four broad strategies to produce novel uses. The four strategies were: using long-term memory to produce ideas for novel uses based on previous experiences, using sensory-perceptual features of the objects (such as having a flat edge) to develop new ideas for use, comparing objects to larger categories of use (such as "weapons" or "transportation"), and mentally disassembling objects to imagine alternative uses for components of the original object. The results from Gilhooly et al. [24] are expanded in a later study that explores the role of cognitive-perceptual re-representation in the Alternative Uses Task, suggesting that participants must mentally or cognitively re-represent common objects based on specific features to produce novel uses [25]. For example, a shoe may be re-represented as something with a hard, flat surface rather than as something to wear, leading a participant to list “hammer” as an alternative use.

Although the AUT is an established measure of divergent thinking, it is not the only measure, nor should it be. One task will never be able to perfectly capture the full concept of divergent thinking. The Alternative Uses Task asks participants to work with items they are already familiar with, requiring participants to overcome fixedness on the traditional use of an object, such as a bowl being used to hold things [26, 27]. While this is an important aspect of creativity, it is not the only aspect–even from a predominantly cognitive-perceptual perspective (and, for the moment, setting aside contributions from other aspects such as motivation or personality). The Alternative Uses Task does not require participants to interpret an ambiguous idea, but rather to work with a tangible object that already exists, and for which they already have an extensive network of semantic and memory-based associations. Yet creativity also includes generating ideas, objects, and products that do not yet exist–including from initial starting points that may be ambiguous, vague, indeterminant, or extremely open-ended [8, 25, 28]. Such conceptual and perceptual ambiguity can itself be an important impetus to creative and design ideation [29], for example, in the process of "seeing as" [30] in which one tries out different properties or attributes of an indeterminant or ambiguous visual-spatial image, to test how those properties mesh with one’s mental representations via metaphors, analogies, or past experiences [31, 32].

The Torrance Tests of Creative Thinking (TTCT) consist of several different divergent thinking tasks [33]. Two of these are primarily verbal in nature and are examined here. The Torrance Product task asks participants to produce ideas to make a children’s toy more fun or marketable. The Torrance Suppose or Consequences task asks participants to imagine what interesting things might occur in a made-up or hypothetical situation, such as if the earth were covered in a thick fog leaving only people’s feet exposed. The Torrance tasks have predictive validity [34]; for example, the Suppose task is correlated with later creative achievement in school and life [35], and divergent thinking scores from the verbal tasks were more predictive of later adult creative achievement than intelligence [36]. These two tasks differ in the underlying processes being used [37], with the Suppose task being relatively more abstract and open-ended than the Product task in which participants are given a more concrete starting point. Both are measures of divergent thinking however, and both are important in understanding the broad range of what divergent thinking means and what assessments of divergent novel ideation can predict.

**Fig 1:**
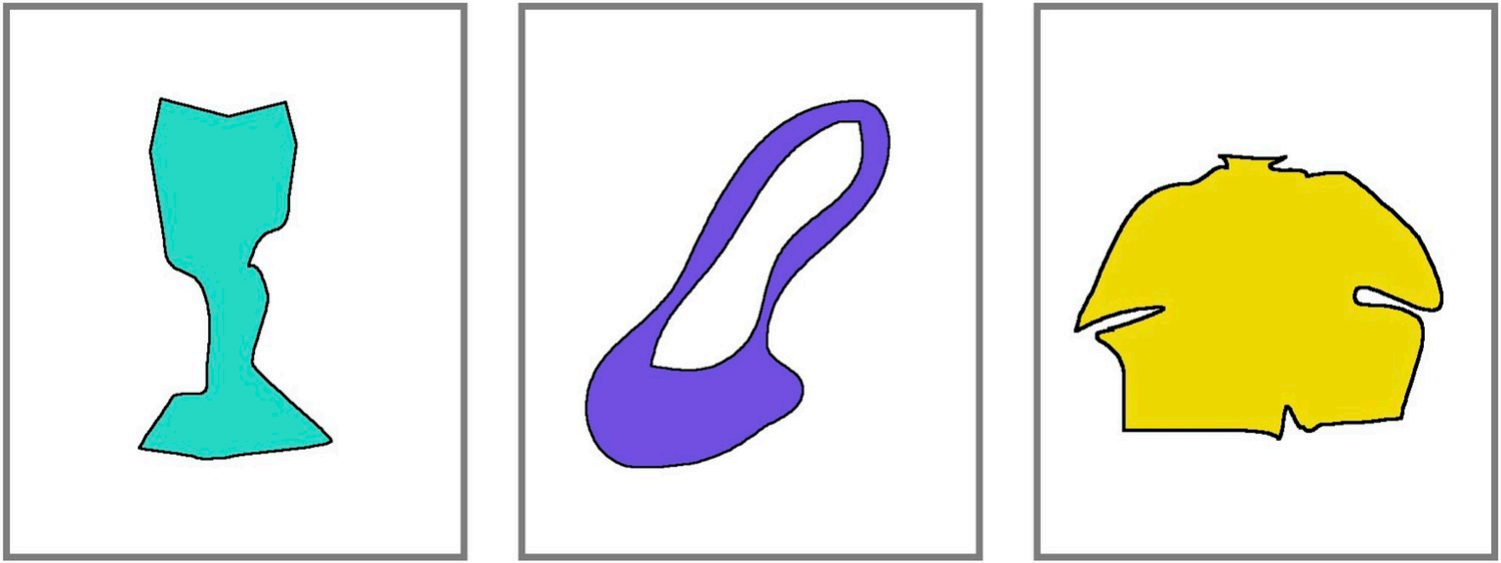
Three illustrative items from the Figural Interpretation Quest.

A newer measure of divergent thinking is the Figural Interpretation Quest (FIQ), which asks participants to produce as many interpretations as possible for a colored ambiguous irregular shape; this task has previously been used in creativity-related research [38], including (as developed further below) specifically in relation to the primary product design ideation [6] outcome measure used in the current study. The different shapes in the FIQ task take varied forms and may have irregular, curved, or straight edges, may be solid or have a space in the middle, and are filled with a solid color (see Methods section, Fig 1 for illustrations). For example, a teal-colored shape that fans out at both ends with a thinner middle may be interpreted as a wine glass, a vase, a shovel, or the head and shoulders of a person looking away into the distance.

Like the Alternative Uses Task, the FIQ asks participants to start with a singular "stimulus" and to mentally represent it (that is, construe or interpret it) in different ways. Although the FIQ has not been as extensively studied as the AUT, previous research using other ambiguous perceptual stimuli such as the Jastrow duck-rabbit or Necker cube bistable images [16–18, 39] or the abstract line-drawn geometrical "Pattern Meanings Test" stimuli created by developmental researchers Wallach and Kogan [40, 41], suggests that flexibility in perceptual interpretation–the ease and frequency with which a stimulus can be construed in alternative ways–is positively correlated with creativity, both as assessed by self-report [39] and by performance on the AUT [39] and Pattern Meanings Test [42]. Additional evidence suggests that openness to experience–the personality trait that has been most robustly and consistently positively associated with creative performance [43, 44]–is positively correlated to performance in bistable image tasks [16] and that attentional focus can impact how an image is perceived in such tasks [18]. More specifically, focusing on a different area of an image can induce perceptual switching between alternative interpretations of an image. It is possible that FIQ performance relies on many of these same processes to produce multiple interpretations for a drawing. These findings support the hypothesis that a ready flexibility in altering one’s perception of ambiguous figures is related to creativity, and that aspects of perceptual reinterpretation may contribute to performance on divergent thinking tasks–and creative design ideation more generally [22, 23].

More recently, our research has explored predictive variables of creative performance–including divergent thinking tasks– on an applied Design Product Ideation task. In the Design Product Ideation task, participants were asked to generate novel product ideas that could make the activities of either urban gardening or outdoor picnics more enjoyable, or that could creatively address potential obstacles to effectively engaging in these activities [6]. Results from the study showed that two different divergent thinking tasks, together with two personality characteristics, and a task-specific factor conjointly best concurrently predicted the originality of participants’ ideas on the Design Product Ideation task. Specifically, the five predictors included originality scores on the Alternative Uses Task, originality scores on the Figural Interpretation Quest, the Openness aspect of Openness to experience, the Industriousness aspect of Conscientiousness, and previous knowledge relating to the Design Product Ideation task (gardening). Multiple regression analyses showed that each of these variables significantly predicted performance, meaning that each is measuring something different enough from the other measures to individually predict creative performance on the Design Product Ideation task. These findings raise the question of what cognitive processing differences exist between the AUT and the FIQ that result in their differential (incremental) predictiveness for generating original ideas in response to the Design Product Ideation prompt.

Table 1 presents an initial possible cognitive task analysis for the four divergent thinking measures (AUT, FIQ, Torrance Suppose, Torrance Product) and the Design Product Ideation task. As can be seen from the table, all four divergent thinking measures likely share a number of cognitive processes with each other that may also contribute to originality of Design Product Ideation, such as abstraction, drawing on past experiences/training, and epistemic curiosity. However, the four divergent tasks also likely differ from one another for other cognitive processes. For instance, conceptual combination, conceptual restructuring, needs and desires, and problem identification all may be less influential in the FIQ than in the remaining divergent tasks, but the FIQ might call upon visuospatial processes such as mental rotation and imagined segmentation of part-whole relations [25, 26] that might be less likely to contribute to the Torrance Suppose task. Note that the listing of cognitive processes and relevant literature in the table is neither meant to be exhaustive nor to be fully definitive, but rather to provide a theoretical and empirical rationale for why the divergent thinking measures might yield different degrees of predictive value for outcome measures such as the Design Product Ideation task.

**Table 1:** Cognitive task analysis of creative processes for the four divergent thinking measures and Design Product Ideation Task.

Process	AUT	FIQ	Torrance Suppose	Torrance Product	Design Product Ideation
**Abstraction**	**Y** ^**a**^	**Y**	**Y**	**Y**	**Y**
Analogical Thinking	Y ^b^	Y	Y	Y	Y
Analysis	Y ^c^	Y	Y	Y	Y
Associative Thinking	Y ^d^	Y	Y	Y	Y
Categorization	Y ^c^	Y	Y	Y	Y
~ Conceptual Combination	Y ^e^	Unclear	Y	Y	Y
~ Conceptual Restructuring	Y ^a^	Unclear	Y	Y	Y
**Divergent Thinking**	**Y** ^c^	**Y** ^**j**^	**Y**	**Y**	**Y**
**Drawing on past experiences/training**	**Y** ^c^	**Y**	**Y**	**Y**	**Y** ^**j**^
**Epistemic Curiosity**	**Y** ^**f**^	**Y** ^**f**^	**Y** ^**f**^	**Y** ^**f**^	**Y** ^**f**^
~ Mental Rotation	Y ^a^	Y ^a^	N	Y	Y
~ Needs and Desires	Y ^c^	N	Y	Y	Y
~ Part-Whole relations	Y ^c^	Y	N	Y	Unclear
**~ Perceptual Curiosity**	**Unclear**	**Y** ^**i**^	**Y** ^**i**^	**Unclear**	**Unclear**
~ Perceptual Restructuring	Y ^a^	Y ^a^	N	N	N
Perspective Taking	Y ^g^	Y	Y	Y	Y
~ Problem Identification	Y ^c^	N	Y	Y	Y
**Verbal Skills / Vocabulary**	**Y** ^**h**^	**Y** ^**h**^	**Y** ^**h**^	**Y** ^**h**^	**Y** ^**h**^

Items in bold are being measured in the present study. Cognitive processes preceded by a tilda (~) indicate instances for which the likely contribution of one or more of the four divergent thinking tasks to originality of Design Product Ideation differed from the others. Selected relevant sources for the designated cognitive processes are indicated with superscript letters as follows.

^a^Olteţeanu et al., 2019 [25].

^b^Jones & Estes, 2015 [45].

^c^Gilhooly et al., 2007 [24].

^d^Benedek et al., 2012 [57].

^e^Olteţeanu & Shu, 2018 [26].

^f^Hardy et al., 2017 [46].

^g^Long & Toppino, 2004 [18].

^h^Laukkonen & Tangen, 2017 [17].

^i^ Litman & Spielberger, 2003 [61].

^j^Tran et al., 2020 [6].

The present study is a conceptual replication and extension of the research reported by Tran et al. [6]. We have three main aims. First, motivated by the findings from the Tran et al. [6] study, we seek to further test the relationship between Design Product Ideation originality scores and various measures of divergent thinking, personality, cognitive ability, task-specific factors, and previous creative experience. The previous study only included a single timepoint of assessment for many of the variables but, to further assess the consistency of findings, the present study assessed the concurrent predictors of originality in Design Product Ideation twice, on two separate occasions termed pre and post in this report.

Second, we seek to systematically evaluate the incremental predictive value for Design Product Ideation originality provided by the four behavioral assessments of divergent thinking over and above several separately entered individual differences, training, and task-specific measures. Specifically, motivated by a componential view of creativity, we examined the degree to which the divergent thinking measures added incremental predictive value for originality on the Design Product Ideation over: (a) *the personality characteristics of openness to experience and curiosity*. From among the five factors in the "big five" taxonomy of personality including Neuroticism, Extraversion, Openness to Experience, Agreeableness, and Conscientiousness [47, 48] Openness to experience is, as noted earlier, the personality trait that has been most robustly and consistently positively associated with creative performance [43, 44]. Curiosity is an aspect that is often linked to openness to experience [48], for instance, individuals high in openness often actively seek out novel and varied activities [49], and curiosity also has been shown, albeit less often, to positively correlate with self-reported and behavioral measures of creativity [50]. (b) *prior formal or informal creativity-related training and activities*. Engagement in varied creativity-related activities can contribute to creative achievement [51] and to what Cropley [52] (p. 397) characterized as knowledge that "indicates which kinds of attack on a problem are likely to be fruitful (or are already known to be fruitless)." (c) *general cognitive ability*, *as reflected in standardized measures of abstract fluid reasoning and vocabulary*. Although the exact nature of the contribution of intelligence or general mental ability to creative thinking continues to be debated [53–55], meta-analyses reveal that there is a modest positive correlation between both fluid and crystallized facets of intelligence with creativity [56]. This correlation partially reflects such general cognitive processes as memory/conceptual retrieval ability [57] though these domain-general abilities likely coexist with sets of domain-specific and task relevant abilities [53]. Accordingly, we also assessed (d) *task-specific factors*, such as knowledge of the task domain for the Design Product Ideation challenge, and interest or engagement in that task.

Third, given the comparative newness of the FIQ as a measure of divergent thinking, we also assess how strongly originality on this perceptually-prompted ambiguous shapes interpretation task is associated with originality of Design Product Ideation when controlling for (that is, partialling out) participants’ scores on other measures that are correlated with Design Product Ideation.

To examine predictors of the originality of Design Product Ideation, the current study initially uses measures from the most predictive multiple regression model from Tran et al. [6] for comparison. As noted above, that model included AUT originality, FIQ originality, Industriousness, Openness to experience, and knowledge of Design Product Ideation at pre-test. Should the same pattern be observed, this suggests that there is consistency in the previous finding that these factors differentially predict creative performance as measured by originality scores on the domain-specific Design Product Ideation task. Following this analysis, other significantly correlated measures will be tested in the predictive model. The previous study did not measure predictiveness of these factors using divergent-thinking and other assessments that were administered at post-test following intervention (rather than only at pre-test), but the present study will do so. It is hypothesized that Design Product Ideation at both pre-test and post-test will be predicted by originality of performance on one or more divergent thinking measures, but will also be conjointly predicted by one or more personality, prior experience, cognitive ability, or task-specific factors.

## Methods

### Experimental design

This study utilized a repeated-measures design, with each of the measures being implemented at pre- and post-intervention. The key dependent variable was originality on the Design Product Ideation at pre- and post-intervention respectively. Independent variables were scores for originality on the AUT, FIQ, and Torrance Suppose and Product tasks, personality aspects of Openness to experience and Industriousness, and perceived challenge and previous knowledge of the Design Product Ideation prompt. There was no control group in this study, as all participants participated in pre- and post-tests as well as the intervention, and the present study is not concerned with course-related improvement in creative performance over time but rather assesses what cognitive-behavioral and individual difference factors predict original ideation on an applied design task at the two timepoints. Participants were given one of two prompts for the Design Product Ideation and AUT at pre-test and the opposite prompt at post-test. (Table 2 provides additional details about the stimuli and task administration.).

**Table 2:** Stimulus and task information.

Measure	Administration Format^a^	Time(s) of Administration	Number of Items^b^
**Creative Performance Measures**			
Design Product Ideation (DPI)	in class	pre- and post-test	1 item at pre-test
	10 min per item		1 item at post-test
Alternative Uses Task (AUT)	in class	pre- and post-test	1 item at pre-test
	5 min per item		1 item at post-test
Figural Interpretation Quest (FIQ)	online	pre- and post-test	4 items at pre-test
	40 sec per item		4 items at post-test
modified Torrance Product	in class	pre- and post-test	1 item at pre-test
	5 min per item		1 item at post-test
modified Torrance Suppose	in class	pre- and post-test	1 item at pre-test
	5 min per item		1 item at post-test
**Individual Difference Measures**			
Design Product Ideation (DPI) Questions^c^	in class	pre- and post-test	5 items at pre-test
			5 items at post-test
Shipley-2			
Abstraction (fluid reasoning)	in class	pre-test	25 items
Vocabulary	in class	pre-test	40 items
Big Five Aspect Scales (BFAS)^d^			
Openness to experience-Openness	online	pre- and post-test	10 items
Openness to experience-Intellect	online	pre- and post-test	10 items
Conscientiousness-Industriousness	online	pre- and post-test	10 items
Curiosity^e^			
Epistemic curiosity	online	pre-test	40 items
Perceptual curiosity	online	pre-test	16 items
Creative Training, Activities, Ideas^f^	online	pre-test	6, 12, 12 items

^**a**^All measures administered in class were given in paper-and-pencil format; all measures administered online were given outside of class time and in digital format.

^**b**^For Design Product Ideation the prompts were picnic at pre-test and urban gardening at post-test; for the AUT the items were paper clip and blanket respectively; for the FIQ, four items were used at pre-test and four different items were used at post-test; for the modified Torrance tasks, Form A and Form B were counterbalanced, with approximately half of the participants receiving Form A at pre-test and Form B at post-test, and the converse for the other participants.

^**c**^The Design Product Ideation (DPI) Questions were administered directly after the DPI task; the 5 items assessed the participants’ knowledge of the DPI topic/subject matter, interest in the DPI topic, enjoyment in the DPI task, engagement in the DPI task, and how challenging they found the DPI task; each item was assessed on a 7-point scale (1 = not at all, 4 = a moderate amount, 7 = a great deal).

^**d**^The full BFAS questionnaire includes 100 items; for current purposes only the two aspects of Openness to experience (Openness and Intellect) and the Industriousness aspect of Conscientiousness, each assessed with 10 items, are considered.

^**e**^The Curiosity items were assessed on a 4-point scale (1 = almost never, 2 = sometimes, 3 = often, 4 = almost always).

^**f**^The Creative Training, Activities, Ideas questionnaire included 6 items assessing creativity-related education/ training, and 12 items assessing the frequency with which participants had engaged in various creative or design activities ("activities"), and whether they often had ideas related to those same areas–whether or not they’d had the opportunity to realize them ("ideas").

### Participants

Participants in this experiment were 99 undergraduate students enrolled in an introductory design class at the University of Minnesota; an additional 13 participants were excluded from data analyses due to missing data on multiple measures. Of the participants included in the study, 53 identified as male, 45 identified as female, and 1 did not identify as male or female. The mean age of participants was 20.08 (SD = 3.61, N = 98), and the mean number of years of education beginning with first grade was 13.84 (SD = 1.62, N = 99). Most participants were native English speakers (N = 93) (defined as speaking the language before 6 years of age), and 6 were not native English speakers. A majority reported having normal or corrected to normal vision (N = 96), while 3 did not.

The design course was an in-person semester-long class through the College of Design at the University of Minnesota. The course was designed to improve creative thinking and performance through a combination of lectures, projects, and hands-on experience. Measures of personality and creativity for the purpose of this study were completed both in the first weeks of the class (referred to as pre-test measures) and final weeks of the class (termed post-test measures). Participants received participation credit in the course for completing the study measures. The study was approved by the University’s Institutional Review Board. Participants provided written informed consent for inclusion in the study.

### Materials

Some of the measures were completed on paper during class, and others were completed online outside of class time. Table 2 provides an overview of the materials, including the method of administration, the number of items, and other details.

#### Design Product Ideation

Participants were given one of two Design Product Ideation tasks asking them to produce as many products as possible to improve the experience of either urban gardening or a picnic. The prompts were identical to those originally developed for, and employed in, the earlier study of Tran et al. [6] and were as follows:

“A local retail store is interested in creating new products for next summer related to picnics. A picnic, in the most general sense, is an occasion involving taking a packed meal to eat outdoors. You are tasked with generating many new product ideas that can enhance a picnic experience.”

“A local company produces outdoor products that you may find at a hardware store. They are interested in breaking into the market of urban gardening. Urban gardening is essentially gardening in indoor or small spaces (a roof deck, a small yard, indoor areas, window sills, walls, ceiling, etc.). You are tasked with generating many new product ideas that can be used for urban gardening.”

In responding, participants could choose to solve existing problems with picnics or urban gardening, focus on aesthetics, strive for sustainability, make these experiences more entertaining, and more. For example, in the picnic prompt, participants could suggest a solution to ants crawling on a picnic blanket, creating biodegradable utensils, or create a more modern design for a picnic basket that involves a Bluetooth speaker.

Responses to the Design Product Ideation task were scored for relevance, originality, and value, but only originality will be considered in the present study. The dependent measure of relevance assesses whether responses are related to the prompt, regardless of novelty. The dependent measure of value assesses whether the suggestion would be marketable, useful, fun, or represent current trends. Originality scores are based on the novelty of an idea, regardless of whether the idea is feasible. Each response received a score of 0 for unoriginal ideas, 1 for somewhat original ideas, and a 2 for very original ideas.

#### Design Product Ideation questions

Task-specific data about the perceived challenge and previous knowledge of the Design Product Ideation topic were collected using self-report measures. Participants were asked to rate on a Likert scale from 1 to 7 how much they were interested in, challenged by, and engaged in the Design Product Ideation, as well as their previous knowledge of the Design Product Ideation topic.

#### Alternative Uses Task

The Alternative Uses Task (AUT) is a divergent thinking task that asks participants to describe as many uses as possible for a common object in a set amount of time [9]. For example, a participant may be asked to name novel uses for a paper clip that do not include the typical use of holding together sheets of paper. Participants’ responses are scored on fluency and originality, although only originality is analyzed in the present study. Fluency is a measure of the number of responses that appropriately answer the prompt, regardless of novelty. Originality measures the novelty of each response. Highly novel responses received a score of 2, somewhat novel responses received a 1, and unoriginal or non-fluent responses received a 0. Originality is the focus of the current work because it measures novel ideas as opposed to reproducing known uses.

#### Figural Interpretation Quest

The Figural Interpretation Quest (FIQ) is a newly developed measure of creativity where participants are asked to produce as many novel explanations or construals as possible for an ambiguous figure [6, 38]. For example, a teal colored shape that fans out at both ends with a thinner middle may be interpreted as a wine glass, a shovel, or the head and shoulders of a person looking away into the distance. The stimuli are a selected subset of items originally developed to examine semantic contributions to episodic memory [58]. Three illustrative items from the FIQ are shown in Fig 1.

For the purpose of the current study, only originality scores are included in analysis, though fluency and flexibility were also evaluated. Fluency measured the number of appropriate and intelligible responses, while flexibility provided a measure of how diverse responses are categorically. For example, responses may be in the categories of an animal, an article of clothing, or geographical features. Originality measured the extent to which "responses were uncommon, novel, striking, fun, or otherwise original." Unoriginal or non-fluent responses received a score of 0 for originality, somewhat original responses were given a 1, and very original responses were given a 2.

#### Torrance task

Two modifications of the Torrance Test of Creative Thinking were used in this study [33, 35]. The Torrance Suppose task asks participants to make predictions about what interesting things may occur in a theoretical situation, such as if a thick layer of fog were to cover the Earth leaving only people’s feet visible. A second measure, the Torrance Product task, asks participants to make improvements to a child’s toy such as a stuffed monkey. For both tasks, responses were scored on fluency and originality, though only originality scores are considered here. Fluency measured the number of responses that are related to the prompt without novelty being taken into consideration. Individual responses were given scores of either a 0 (unoriginal) or 1 (original). A list of unoriginal responses was used to create consistency in scoring.

#### Creative training, activities, ideas

Previous creative experiences were evaluated using self-report measures. Participants recalled how often they had engaged in creative experiences in four categories. The first is formal creative training (high school classes, college classes, online classes/tutorials). The second is informal creative training, such as internships (< 3 months), employment (> 3 months), or extracurricular activity. Participants indicated "the number of times" they had participated in each of these formal and informal training activities using 7 response options, successively labeled from "0" to "6+" times. The third category assessed participants’ prior creative activities, in the past 5 years, in 12 different domains (literature/writing, music, home-based arts and crafts, products/consumer goods, food and drink, sports, visual arts, performing arts, science, engineering/architecture, technology and information, societal or cultural contributions). The fourth and final category asked participants to report how often, in the past 5 years, they had experienced creative thoughts but–given limits on their time, energy, or other resources–were unable to act on those ideas, such as innovative ideas for how to solve a problem that have not yet been carried out. Participants responded to this unrealized "ideas" question for the same 12 domains as for the third category of creative activities. Responses to the creative activities and creative ideas questions were given on a 1-to-5 scale anchored by "never" and "very often." Scores were calculated after subtracting 1 so that numerical values of zero corresponded to responses of "never."

#### Shipley-2

Shipley-2 measures of vocabulary and fluid reasoning are intended to evaluate an individual’s vocabulary level and logical or fluid reasoning ability [59]. The vocabulary measure consists of multiple-choice questions about the meaning of various words. Because the measures of creativity in this study involve writing out verbal responses, vocabulary differences may be relevant in performance on these measures. The fluid reasoning measure asks participants to fill in a blank space with a response that fits an initial patterned series of words, numbers, or letters. For example: “up, down, east, west, forward, ______”, in which the correct answer would be “backward”. Fluid reasoning may also be relevant in creativity measures in the present study, as participants must make "on-the-spot" or impromptu relational connections between the prompt they are presented with and their previous knowledge.

#### Big Five Aspect Scales

Personality was measured using the Big Five Aspects Scale (BFAS), a self-report measure that asks participants to rank their agreement from 1–5 based on how much they feel various statements describe them [60]. In this study, only Openness to experience and the Industriousness aspect of Conscientiousness were included. Openness to experience can be broken down into two facets–Openness and Intellect; both facets reflect a general predisposition to flexibly approach novel ideas, with the Intellect facet involving an emphasis on reason and truth, and the Openness facet emphasizing aesthetics and beauty.

#### Curiosity

Epistemic and Perceptual Curiosity were examined using the Epistemic and Perceptual Curiosity Scale [61]. Epistemic curiosity is defined as a drive for knowledge such as through solving puzzles or filling in knowledge gaps, while Perceptual Curiosity is driven by increased stimulation of any of the senses. Participants were asked to rate their interest in particular situations, such as exploring new places or learning new problem-solving strategies, on a scale of 1 to 4 based on how they "generally feel". A response of 1 indicated "almost never" and a response of 4 indicates "almost always" interested or engaged in various activities related to curiosity.

## Results

### Overview

Our first aim was to further test the relationship between Design Product Ideation originality scores and various measures of divergent thinking, personality, cognitive ability, task-specific factors, and previous creative experience. To address this aim, the present study used results from the Tran et al. [6] study to constrain the measures evaluated, resulting in analysis of originality scores on AUT and FIQ, Openness to experience and the Industrious aspect of Conscientiousness, and the perceived challenge and previous knowledge of the Design Product Ideation topic. These measures were evaluated at both pre- and post-intervention and compared to results from the Tran et al. [6] study, which only included measurements from pre-test.

First, simple (zero-order) correlations between each of the potential predictive factors and Design Product Ideation originality scores were determined using Pearson’s correlations. Based on these correlations, four more variables were added into the multiple regression model. These measures included: previous formal creative training or education, previous informal training or education, and originality scores for Torrance Suppose and Torrance Product tasks.

### Inter-rater reliability

All creative performance tasks (Design Product Ideation, AUT, FIQ, Torrance Product, and Torrance Suppose) were assessed by two independent raters, who were blind to condition (pre-test or post-test). Each item (e.g., Torrance Product with the toy monkey) was assessed for fluency, originality, and (if applicable) flexibility and value. Results for the inter-rater reliability are presented in Table 3. As can be seen from the table, strong inter-rater reliability of at least .80 was observed for all measures, with inter-rater correlations between .86 and .92 for originality.

**Table 3:** Inter-rater reliability of the creative performance measures.

Measure	Inter-rater Reliability	
	Pre-test	Post-test
Design Product Ideation (DPI)^a^		
Relevance/Fluency	.98	.94
Value	.99	.96
Originality	.86	.86
Alternative Uses Task (AUT)^b^		
Fluency	.94	.98
Originality	.84	.88
Figural Interpretation Quest (FIQ)^c^		
Fluency	.91	.99
Flexibility	.90	.91
Originality	.87	.86
**Across Pre-Post** ^d^		
modified Torrance Product, Form A		
Fluency	.95	
Flexibility	.80	
Originality	.87	
modified Torrance Product, Form B		
Fluency	.98	
Flexibility	.88	
	.86	
modified Torrance Suppose, Form A		
Fluency	.99	
Originality	.92	
modified Torrance Suppose, Form B		
Fluency	.98	
Originality	.92	

^a^For Design Product Ideation, *N* = 98 at pre-test, *N* = 87 at post-test.

^b^for AUT, *N* = 99 at pre-test, *N* = 97 at post-test.

^c^for FIQ, *N* = 98 at pre-test, *N* = 99 at post-test.

^d^For modified Torrance Product and for modified Torrance Suppose, *N* = 99 at pre-test, *N* = 98 at post-test; inter-rater reliability for the modified Torrance tasks is reported for the separate forms (Form A or Form B), which were counterbalanced across pre-test and post-test.

Table 4 presents the descriptive statistics for these same creative performance measures (Design Product Ideation, AUT, FIQ, Torrance Product, and Torrance Suppose), while Table 5 presents descriptive statistics for each of the individual difference measures (knowledge of, interest in, enjoyment of, engagement in, and challenge of Design Product Ideation task; Shipley-2 Abstraction and Vocabulary tasks; the Industrious aspect of Conscientiousness, Openness and Intellect aspects of Openness to experience as measured by the BFAS; Epistemic and Perceptual Curiosity scores; and prior creative training, activities and ideas).

**Table 4:** Descriptive statistics for the creative performance measures.

Measure	Pre-test		Post-test	
	Mean	95% CI	Mean	95% CI
Design Product Ideation (DPI)^a^				
Relevance/Fluency	32.44	30.05–34.83	31.03	28.49–33.57
Originality	17.37	15.72–19.02	15.45	14.01–16.88
Value	32.81	30.27–35.34	32.39	29.77–35.00
Alternative Uses Task (AUT)^b^				
Fluency	14.85	13.93–15.78	19.15	18.29–20.02
Originality	7.85	7.28–8.43	18.53	17.67–19.38
Figural Interpretation Quest (FIQ)^c^				
Fluency	4.95	4.66–5.24	5.51	5.15–5.87
Flexibility	3.92	3.73–4.11	4.18	3.94–4.41
Originality	1.42	1.24–1.59	1.85	1.63–2.07
modified Torrance Product^d^				
Fluency	11.03	10.03–12.02	13.66	12.64–14.69
Flexibility	6.35	5.96–6.74	8.43	7.97–8.89
Originality	6.78	6.14–7.43	9.46	8.71–10.22
modified Torrance Suppose				
Fluency	11.71	10.90–12.51	12.79	11.80–13.78
Originality	8.48	7.88–9.08	8.59	7.90–9.27

^a^ For Design Product Ideation, *N* = 98 at pre-test, *N* = 87 at post-test.

^b^ For AUT, *N* = 99 at pre-test, *N* = 97 at post-test.

^c^ For FIQ, *N* = 98 at pre-test, *N* = 99 at post-test.

^d^ For modified Torrance Product and for modified Torrance Suppose, *N* = 99 at pre-test, *N* = 98 at post-test.

**Table 5:** Descriptive statistics for the individual difference measures.

Measure	Pre-test		Post-test	
	Mean	95% CI	Mean	95% CI
Design Product Ideation (DPI) Questions^a^				
Knowledge of DPI topic	4.15	3.88–4.43	3.22	2.89–3.55
Interest in DPI topic	4.16	3.88–4.44	4.18	3.83–4.54
Enjoyment in DPI task	4.56	4.31–4.81	4.09	3.80–4.38
Engagement in DPI task	4.92	4.66–5.18	4.18	3.86–4.51
Challenge in the DPI task	4.88	4.64–5.11	4.80	4.56–5.05
Shipley-2^b^				
Abstraction (fluid reasoning)	111.53	109.53–113.52		
Vocabulary	110.95	109.34–112.55		
Big Five Aspect Scales (BFAS)				
Openness to experience- Openness	3.91	3.80–4.02	3.89	3.78–4.00
Openness to experience-Intellect	3.57	3.44–3.69	3.52	3.40–3.64
Conscientiousness-Industriousness	3.06	2.97–3.14	3.00	2.90–3.10
Curiosity^d^				
Epistemic curiosity	2.97	2.88–3.06		
Perceptual curiosity	3.00	2.90–3.09		
Creative Training, Activities, Ideas^e^				
Formal creative training	9.75	8.88–10.62		
Informal creative training	10.77	9.55–11.98		
Activities	30.43	28.89–31.98		
(Unrealized) Ideas	31.89	30.25–33.53		

^a^The Design Product Ideation (DPI) Questions were administered directly after the DPI task; *N* = 98 for pre-test, *N* = 87 for post-test; each item was assessed on a 7-point scale (1 = not at all, 4 = a moderate amount, 7 = a great deal).

^b^The Shipley-2 scores are age-standardized scores, *N* = 97. This measure was administered at pre-test only.

^c^For the BFAS measures, *N* = 97 and *N* = 99 for pre-test and post-test respectively; each item was assessed on a 5-point scale (1 = strongly disagree, 3 = neither agree nor disagree, 5 = strongly agree).

^d^For the Curiosity measures, *N* = 99; each item was assessed on a 4-point scale (1 = almost never, 2 = sometimes, 3 = often, 4 = almost always). This measure was administered at pre-test only.

^e^For the Creative Training, Activities, Ideas measure, *N* = 99; the average sum of formal creative training, informal creative training, creativity-related activities, or (unrealized) creative ideas is provided. This measure was administered at pre-test only.

### Correlations of measures with Design Product Ideation originality

Table 6 provides a summary of correlations of creative performance and individual differences measures with Design Product Ideation originality. Correlations are provided at pre- and post-test in the present study, as well as, for comparison, the pre-test correlations from the Tran et al. [6] study (designated as 2020 Pre-test).

**Table 6:** Correlations with Design Product Ideation originality.

Type of Measure	Measure	2020 Pre-test		Pre-test		Post-test	
		*r*	95% CI	*r*	95% CI	*r*	95% CI
Creative Performance	AUT originality	.38***	.20 –.54	.50***	.34 –.64	.32**	.11 –.50
Creative Performance	FIQ originality	.32**	.13 –.48	.34***	.16 –.51	.43***	.24 –.58
Creative Performance	modified Torrance Suppose originality	.13	-.07 –.32	.64***	.51 –.75	.45***	.26 –.60
Creative Performance	modified Torrance Product originality	.15	-.05 –.34	.55***	.39 –.67	.52***	.34 –.66
Personality Aspect	Openness-Openness	.16	-.04 –.35	.25*	.06 –.43	.39***	.19 –.55
Personality Aspect	Openness-Intellect	.21*	.02 –.40	.24*	.04 –.42	.41***	.21 –.57
Personality Aspect	Conscientiousness-Industriousness	.22*	.02 –.40	-.04	-.24 –.16	.11	-.10 –.32
Personality Aspect	Epistemic Curiosity	-.04^a^	-.24 –.16	.04	-.17 –.23	.03	-.18 –.24
Personality Aspect	Perceptual Curiosity	-.04^a^	-.24 –.16	.09	-.11 –.29	.13	-.08 –.33
Task Specific (self-report)	Design Product Ideation Knowledge	.28**	.08 –.45	.02	-.18 –.22	.24*	.03 –.43
Task Specific (self-report)	Design Product Ideation Challenge	-.34***	-.50 –-.15	-.31**	-.48 –-.12	-.34***	-.51 –-.14
Prior Creative Experience	Formal Creative Training/Education	--	--	.21*	.01 –.39	.23*	.02 –.42
Prior Creative Experience	Informal Creative Training/Education	--	--	.33***	.14 –.50	.18	-.04 –.37
Prior Creative Experience	Creativity-Related Activities	--	--	.01	-.19 –.20	-.02	-.23 –.19
Prior Creative Experience	Unrealized Creative Ideas	--	--	-.06	-.26 –.14	-.09	-.30 –.12
Cognitive Ability	Abstraction/Fluid Reasoning	.15	-.05 –.34	.16	-.04 –.35	.04	-.17 –.25
Cognitive Ability	Vocabulary	--	--	.05	-.16 –.24	-.03	-.24 –.18

^a^For the Tran et al. [6] study, Epistemic and Perceptual Curiosity were combined into a single Curiosity score.

—Indicates the measure was not administered.

*** *p* < .001

** *p* < .01

* *p* < .05.

Focusing first on the current study’s findings for pre-test, significant positive correlations are seen with Design Product ideation originality scores for each of the four creative performance divergent thinking measures, as well as for Openness and Intellect facets of Openness to experience, perceived challenge of the Design Product Ideation task, and self-reported formal and informal creative training. When compared to results from Tran et al. [6], Torrance Suppose and Product as well as the Openness aspect of Openness to experience show significant correlations not seen in the previous study. The Industriousness aspect of Conscientiousness and previous knowledge of the Design Product Ideation topic were significant in Tran et al. [6] but are not significant in the present study. The two currently observed significant correlations with prior creative training (formal and informal) are from additional variables that were not measured in the 2020 study.

Turning next to the patterns of correlations observed at post-test, correlations with Design Product Ideation originality are again seen for each of the four divergent thinking measures. The Openness and Intellect aspects of Openness to experience are also significantly correlated with originality scores on the DPI task. The task-specific factors of previous knowledge of the topic of the Design Product Ideation task as well as perceived challenge in completing the DPI are significantly related to DPI originality at post-test, a departure from the current findings at pre-test where challenge was the only significant task-specific factor significantly correlated with DPI performance. Formal creative training is significantly associated with DPI originality at both pre-test and post-test, but informal creative training, though still positively associated, no longer shows a significant correlation with DPI originality at post-test.

#### Creativity-related training

Formal creativity training (in school or online classes) was reported by all participants, *M* = 9.75, *SD* = 4.36. The minimum number of formal training experiences was 3 and the maximum was 21. For informal training (such as through work or other organizations), participants reported a minimum of 14 and a maximum of 53 experiences, *M* = 31.89, *SD* = 8.21. When asked about engagement in other creative activities such as writing, science, or music, participants reported a minimum of 14 and a maximum of 53 experiences, *M* = 30.43, *SD* = 7.77. Similarly, when asked about unfulfilled creative ideas in these varied domains, participants reported a minimum of 14 and a maximum of 53 ideas, *M* = 31.89, *SD* = 8.21.

#### Curiosity

On self-reported curiosity measures, the mean score (on a 4-point scale) for Epistemic Curiosity was 2.97, *SD* = .46, *N* = 99. The mean score for Perceptual Curiosity was 3.00, *SD* = .48, *N* = 99. Although Perceptual and Epistemic Curiosity were significantly correlated with each other (*r* = .67, *p* < .001), neither was significantly correlated with any of the originality measures. It might be noted, however, that whereas the correlation with Epistemic Curiosity was near zero, Perceptual Curiosity showed a slightly numerically higher correlation to originality scores at both pre-test (*r* = .09) and post-test (*r* = .13). These findings suggest that curiosity–at least as measured by the questionnaires used in this study–did not play a significant role in Design Product Ideation performance.

#### Shipley-2 Abstraction and Vocabulary

In analysis of the Shipley-2 Abstraction and Vocabulary, scores were standardized by age with a mean of 100 and a standard deviation of 15. For *N* = 97, the mean Shipley-2 Abstraction score was 112 with a standard deviation of 9.89 and the mean for Shipley-2 Vocabulary was 111 with a standard deviation of 7.96. Based on age-based standardization norms, these sample means for both Abstraction and Vocabulary fall into the “above average” range (110–119).

Considering these two cognitive ability measures, there is no significant correlation between either Shipley-2 Abstraction, or Shipley-2 Vocabulary, with either pre-test originality scores or post-test originality scores for the Design Product Ideation.

### Aim 1: Multiple regression analyses predicting originality of Design Product Ideation

Before conducting multiple regression analyses, we considered pairwise correlations of each of the variables with Design Product Ideation originality scores. A summary of the correlations can be found in Table 6. As can be seen from Table 6, results show a significant positive correlation with formal creative experience (*r* = .21), informal creative experience (*r* = .33), Torrance Suppose originality (*r* = .64) and Torrance Product originality (*r* = .55) scores. There was a significant negative correlation found between Design Product Ideation originality and perceived challenge of Design Product Ideation (*r* = -.31). Prior creative experiences were not reported in the previous 2020 study, and originality scores on Torrance Suppose and Product tasks were not previously found to be significantly correlated to Design Product Ideation performance. Although Design Product Ideation knowledge and the Industriousness aspect of Conscientiousness were correlated with Design Product Ideation originality in the previous study, there is no significant correlation in the present study at pre-test. (However, it may be that perceived challenge in responding to the Design Product Ideation–which here was significantly negatively predictive of Design Product Ideation originality–is, in part, indirectly indexing a lack of Design Product Ideation knowledge.)

We first considered what factors predicted Design Product Ideation originality at pre-test. When entering the pre-test predictors from the Tran et al. [6] study (AUT originality, FIQ originality, Openness to experience (combined aspects), Design Product Ideation knowledge, and the Industriousness aspect of Conscientiousness), AUT originality (*t* = 4.18, *p* < .001) and FIQ originality (*t* = 2.46, *p* = .016) scores were the only significant predictors of Design Product Ideation originality at pre-test in the current study. We therefore excluded the nonsignificant predictors from the prior study and separately entered the newly identified correlates of DPI originality, including the Torrance measures and creativity training related measures.

When compared to the initial model for pre-test using only AUT originality, FIQ originality, and perceived challenge of the Design Product Ideation, formal creativity training does not improve the predictive model. Informal creative training has marginal predictive validity (standardized Beta = .14, *t* = 1.61, *p* = .11). This provides only a slight improvement from Adjusted R^2^ = .38 in the previous model to Adjusted R^2^ = .39 in the current model. When Torrance Suppose from pre-test is added, it becomes a significant predictor, and each of the other retained predictors remain significant. The final Adjusted R^2^ = .50 for the model including Torrance Suppose at pre-test. When adding Torrance Product originality at pre-test, it too becomes a significant predictor and each of the other retained predictors remain significant. The adjusted R^2^ = .45 for this model.

Based on these analyses, the most predictive model for Design Product Ideation originality at pre-test includes AUT originality, FIQ originality, perceived challenge of Design Product Ideation, and Torrance Suppose originality scores from pre-test. The final model is *F*(4, 92) = 24.85, *p* < .001 with an adjusted R^2^ = .50. A summary of the multiple regression on Design Product Ideation originality at pre-test is included in Panel A of Table 7.

**Table 7:** Multiple regression on Design Product Ideation originality.

**A. Design Product Ideation originality at pre-test, adjusted R**^**2**^ **= .50**							
Source	*B*	*SE B*	β	*t*	*p*	95% CI for B	
						lower	upper
(Constant)	8.03	3.48		2.31	.023	1.11	14.94
PreAUT originality	.52	.20	.22	2.60	.011	.12	.92
PreFIQ originality	1.59	.75	.17	2.13	.036	.11	3.07
PreDPI challenge	-1.73	.52	-.25	-3.30	.001	-2.76	-.69
PreSuppose originality	1.17	.24	.43	4.82	< .001	.69	1.65
**B. Design Product Ideation originality at post-test, adjusted R**^**2**^ **= .39**							
Source	*B*	*SE B*	β	*t*	*p*	95% CI for B	
						lower	upper
(Constant)	-8.23	4.62		-1.78	.079	-17.43	.96
PostFIQ originality	1.36	.65	.20	2.09	.04	.07	2.66
PostOpenness	1.99	.67	.28	2.96	.004	.65	3.32
PostProduct originality	.67	.16	.38	4.20	< .001	.35	.99

We next considered what factors predicted Design Product Ideation originality at post-test. Using the most predictive model from the Tran et al. [6] pre-test (that is, AUT originality, FIQ originality, Openness to experience, Industriousness, and knowledge of Design Product Ideation topic) results from the present study at post-test showed that Industriousness and knowledge of the topic of the Design Product Ideation were not significant predictors. Excluding these two nonsignificant predictors yielded a regression model of *F*(3, 81) = 12.45, *p* < .001 with three significant predictors: Post-AUT originality (standardized Beta = .21, *t* = 2.21, *p* = .03), post-FIQ originality (standardized Beta = .26, *t* = 2.63, *p* = .01), and post-Openness (standardized Beta = .31, *t* = 3.00, *p* = .004), Adjusted R^2^ = .29.

Based on significant correlations from the post-test measures shown in Table 6, formal creative training, informal creative training, and Torrance Suppose and Product originality scores were separately tested in the model. Adding formal creative training shows that formal creative experience is a significant predictor, and all previous predictors remain significant (Adjusted R^2^ = .33). Informal creative training was not included in the post Design Product Ideation model as the simple correlation was only marginally significant. When Torrance Suppose originality scores at post-test are added to the model, it is only marginally significant (*p* = .102) and makes AUT originality insignificant while only improving the Adjusted R^2^ to .31. Adding Torrance Product originality at post shows it is a significant predictor, but AUT is no longer a significant predictor in the resulting model (Adjusted R^2^ = .37). When AUT originality at post-test is removed from the model, the most predictive model for Design Product Ideation originality at post-test is *F*(3, 82) = 19.07, *p* < .001 with three significant predictors; FIQ originality, Openness to experience, and Torrance Product originality scores (R^2^ = .39). A summary of the regression analysis from post-test is included in Panel B of Table 7.

In summary, based on the most predictive models, Design Product Ideation originality at pre-test was predicted by AUT originality, FIQ originality, perceived challenge of Design Product Ideation, and Torrance Suppose originality scores. At post-test, the most predictive model includes FIQ originality, Openness to experience, and Torrance Product originality scores.

### Aim 2: Evaluating incremental predictive value for DPI originality of other factors

Our second aim was to systematically evaluate the incremental predictive value for Design Product Ideation originality of the four behavioral assessments of divergent thinking over and above individually entered measures of personality (Openness to experience, Curiosity), prior formal or informal creativity-related training, cognitive ability (Shipley-2 Abstraction and Shipley-2 Vocabulary), and task-specific factors such as knowledge of the task domain. Examination of the intercorrelations of the five task-specific DPI questions (knowledge of the DPI topic, interest in the DPI topic, enjoyment in the DPI task, engagement in the DPI task, challenge in the DPI task) revealed that three of the questions: participants’ interest, enjoyment, and engagement in the DPI task were strongly intercorrelated both at pre-test (average pairwise correlation = .64) and at post-test (average pairwise correlation = .63); we therefore first *z*-scored each of these measures, and then combined them into a single measure (termed interest-enjoy-engage).

Table 8 presents a summary of the separate linear regression analyses performed for Aim 2. Specifically, Table 8 provides the change in the proportion of variance accounted for (change in R^2^), for predicting the outcome of originality on Design Product Ideation, for each of the four divergent thinking tasks, at pre-test, and post-test, and in combination with each of the individual difference, training, and task-specific factors. The change in R^2^ for the divergent thinking task is indicated in the first row of each cell for the correspondingly titled columns and is significant (*p* ≤ .05) in all instances. The value in the second row for these same cells indicates any incremental predictive value provided by the designated variable for that row, over and above that provided by the designated divergent thinking task, "—" indicates no significant incremental value for that row variable, using Forward entry, and criterion probability of *F* to enter of *p* ≤ .05. For example, FIQ originality at post-test ("Post FIQ") explained 21% of the variance in originality of post-test Design Product Ideation (first row of that cell); adding the post-assessment of openness to experience to the regression model explained an additional 7% of the variance in originality of post-test Design Product Ideation.

**Table 8:** Incremental predictive value (change in R^2^) for originality of Design Product Ideation.

Variable	Predictive at Pre^a^	Pre AUT	Pre FIQ	Pre Suppose	Pre Product	Predictive at Post ^a^	Post AUT	Post FIQ	Post Suppose	Post Product
Openness to experience		.25	.12	.42	.29		.21	.21	.22	.27
	Weak, 1/4	--	.05	--	--	Yes, 4/4	.05	.07	.08	.11
Epistemic curiosity		.25	.12	.42	.31		.10	.19	.20	.27
	No, 0/4	--	--	--	--	No, 0/4	--	--	--	--
Perceptual curiosity		.26	.12	.43	.30		.11	.20	.20	.27
	No, 0/4	--	--	--	--	No, 0/4	--	--	--	--
Formal training		.25	.12	.41	.30		.10	.18	.20	.27
	Mixed, 2/4	--	--	.05	.06	Mixed, 3/4	.05	.06	.06	--
Informal training		.25	.12	.41	.30		.10	.18	.20	.27
	Yes, 4/4	.06	.07	.07	.08	Mixed, 2/4	--	.04	.04	--
Abstraction (fluid reason.)		.25	.12	.41	.30		.10	.18	.20	.27
	No, 0/4	--	--	--	--	No, 0/4	--	--	--	--
Vocabulary		.25	.12	.41	.30		.10	.18	.20	.27
	No, 0/4	--	--	--	--	No, 0/4	--	--	--	--
DPI Challenge		.25	.12	.41	.30		.11	.18	.20	.27
	Yes, 4/4	.08	.12	.04	.07	Mixed, 3/4	.06	.06	.05	--
DPI Knowledge		.25	.12	.41	.30		.10	.18	.20	.27
	No, 0/4	--	--	--	--	Mixed, 2/4	--	.05	--	.04
DPI Interest-Enjoy-Engage		.25	.12	.41	.30		.10	.18	.20	.27
	Weak, 1/4	--	--	.03	--	No, 0/4	--	--	--	--

The change in proportion of variance accounted for (change in R^2^), for predicting the outcome of originality on Design Product Ideation, for each of the four divergent thinking tasks, at pre-test, and post-test, and in combination with each of the individual difference, training, and task-specific factors. The change in R^2^ for the divergent thinking task is indicated in the first row of each cell for the correspondingly titled columns and is significant in all instances. The value in the second row for these same cells indicates any incremental predictive value provided by the designated variable for that row, over and above that provided by the designated divergent thinking task

—Indicates no significant incremental value for that row variable, using Forward entry, and criterion probability of *F* to enter of *p* ≤ .05.

^a^The columns titled "Predictive at Pre" and "Predictive at Post" summarize the evidence that a given factor (row variable) added incremental predictive value for Design Product Ideation at Pre and Post respectively, with the classifications of "Yes"–the relevant factor added predictive value for all four (4/4) of the divergent thinking tasks, "Mixed"–the factor added predictive value for two (2/4) or three (3/4) of the divergent tasks, "Weak"–the factor added predictive value for only one of the four (1/4) divergent tasks, and "No"–the row variable did not add incremental predictive value for any of the four (0/4) divergent tasks given at either pre-test or post-test.

### Aim 3: A more focused evaluation of the predictive value of FIQ originality

As reported above, original responses on the FIQ were a significant predictor of participants’ originality of Design Product Ideation at both pre-test and post-test. Given that the FIQ is a comparatively new measure of divergent thinking, we also took a more focused, pairwise look at FIQ originality scores when statistically separately controlling for each of the four variables that were found to be correlated with Design Product Ideation. Table 9 presents the zero-order and partial correlations from these analyses.

**Table 9:** Partial correlation analyses for FIQ originality with Design Product Ideation.

Measures	Zero-order correlation	Partial correlation	Measure controlled for
preFIQ–preDPI Originality	.34***	.32***	Formal Creative Training/Education
preFIQ–preDPI Originality	.34***	.30***	Informal Creative Training/Education
postFIQ–postDPI Originality	.42***	.45***	Formal Creative Training/Education
postFIQ–postDPI Originality	.42***	.44***	Informal Creative Training/Education
preFIQ–preDPI Originality	.34***	.18 ^^^	Torrance pre-Suppose Originality
preFIQ–preDPI Originality	.34***	.28**	Torrance pre-Product Originality
postFIQ–postDPI Originality	.42***	.32**	Torrance post-Suppose Originality
postFIQ–postDPI Originality	.42***	.33**	Torrance post-Product Originality

*** p < .001

** p < .01

* p < .05

*^ p* < .10.

From Table 9, it is seen that correlations of FIQ originality with Design Product Ideation are essentially unchanged when accounting for formal or informal creative training/education. In contrast, after accounting for either the Torrance Suppose or Torrance Product originality scores, there is some attenuation of the FIQ-Design Product Ideation correlation, with a similar attenuation found for both the pre-test and post-test comparisons. These attenuations suggest that at least some of the cognitive-perceptual or behavioral-motivational factors that contribute to the relation between FIQ originality and Design Product Ideation are also assessed with the two verbal Torrance tasks. However, the observation that the partial correlations themselves remain comparatively strong, and in three of the four cases statistically significant, suggests that the FIQ is also assessing something more–that is, something over and above–what is assessed by either of the two Torrance tasks.

## Discussion

Based on the outcomes of this study, it is clear that there is no singular measure that is sufficient to fully predict originality scores for a Design Product Ideation task. Despite the heavy reliance on AUT in creativity research, including in cognitive neuroscience studies of creativity [62–64], the present study found that although AUT originality was predictive of Design Product Ideation performance at pre-test, it was not significantly predictive at post-test when other predictive factors were included in a multiple regression model. In contrast, FIQ originality was predictive of Design Product Ideation originality at both pre- and post-test. It is also important to note that despite FIQ’s predictiveness at both pre- and post-test, it is not the only factor contributing to performance. Individual difference measures, task-specific factors, and other creative performance measures were also predictive. Though using a singular measure of divergent thinking to predict creative performance may simplify research methods and analyses, it is unlikely to capture all the underlying cognitive and perceptual processes–many of which we articulated in Table 1 –involved in creative performance on a Design Product Ideation task or other real world creative tasks. This finding aligns with that of earlier studies demonstrating the incremental predictive value of incorporating both conceptual and perceptual divergent thinking assessments in predicting creative problem solving in specific domains such as architectural design [21], and with systematic analyses of conceptual design cognition [31, 32] shown to encompass both multiple cognitive processes (e.g., semantic categorization, association, and retrieval), and multiple visual-perceptual processes (e.g., visual attention, perceptual (re)organization).

Outside of divergent thinking measures, there were other factors that were significantly correlated with Design Product Ideation originality scores. Formal previous creative training or education showed a significant positive correlation at both pre- and post-test. Previous informal creativity training was significantly positively correlated at pre-test and marginally significantly correlated at post-test. This suggests that prior creative training, whether formal or informal, can be effective in improving creative performance on a real-world design task. Support for the potential contributions of prior creativity-related training to Design Product Ideation originality scores was further bolstered by the observation that formal and informal training provided incremental predictive validity–over and above behavioral assessments of originality on our four divergent thinking tasks–at both pre-test and post-test (Table 8). At the outset of the class, informal training, in particular, added significant incremental validity for all four divergent thinking measures and (across pre and post) did so for 6 of the 8 measurement occasions. Formal training added significant incremental validity in 5 of the 8 measurement occasions. This is an important finding, as it supports the idea that creative practice and training have the potential to improve real-world creative performance.

Two task-specific measures were also significantly correlated to Design Product Ideation performance. First, the perceived challenge of the Design Product Ideation task showed a significant negative correlation at both pre- and post-test, a finding also seen at pre-test in Tran et al. [6]. This finding suggests that as a task becomes excessively challenging, participants perform at lower levels with respect to originality. Previous knowledge of the topic of the Design Product Ideation task was significantly positively correlated with originality scores at post-test, but not at pre-test in the current study, while a significant positive correlation was seen in Tran et al. [6]. It is unclear why knowledge of the prompt was not significant at pre-test in the current study. One possibility is that there was a different distribution of participant background knowledge regarding the two design product ideation topics (e.g., the mean responses suggest that participants had more previous knowledge of picnics than of gardening). Overall, across the different task-specific measures, perceived challenge of the Design Product Ideation task added the most incremental predictive validity for originality on that ideation task, adding significant incremental validity for 7 of the 8 pre-and-post measurement occasions. In contrast, the combined measure of interest in, enjoyment of, and engagement with the Design Product Ideation task was seldom predictive over and above our behavioral divergent thinking measures.

Openness to experience showed a significant positive correlation to Design Product Ideation performance at both pre- and post-test, supporting the correlation seen in Tran et al. [6]. Additionally, at post-test, Openness to experience added significant incremental validity–over and above behaviorally-based assessments of originality–on all four of the divergent thinking tasks (changes in R^2^ of .05, .07, .08, and .11 for AUT, FIQ, Suppose, and Product respectively). The positive correlations of Openness to experience with Design Product Ideation at both pre-test and post-test, and the especially marked predictive power of this cognitive-motivational predisposition at post-test, suggests that Openness to experience reliably predicts originality scores on the Design Product Ideation task. Together, these findings should encourage educators and employers to provide interventions or work contexts that support the forms of exploration, risk-taking, and welcoming receptivity to novel ways of doing and thinking that are characteristic of individuals high in Openness to experience [4, 5] and that–by fostering intrinsic motivation and creative process engagement–can heighten creativity [65].

The previous study also found a significant correlation at pre-test between the Industriousness facet of Conscientiousness and Design Product Ideation originality, but that finding was not replicated in the present study. The reason for the lack of predictive value of this personality characteristic in the present study is also unclear; however, it is possible that the contribution of Industriousness is more influential when participants have less prior knowledge. In line with this speculation, somewhat stronger correlations of Industriousness with DPI performance were found for the two occasions when the Design Product Ideation prompt involved gardening, for which participants also self-reported less prior knowledge as compared to when the prompt involved picnics.

Our third aim, given that the FIQ is a comparatively new measure of divergent thinking, was to take a more focused, pairwise look at FIQ originality scores when statistically separately controlling for each of the variables that were found to be correlated with Design Product Ideation. Based on partial correlation analyses between FIQ originality and Design Product Ideation originality, it seems that the FIQ-DPI relationship is minimally affected by previous creative training. In contrast, Torrance Product and Torrance Suppose originality scores do account for some of the correlation between FIQ and DPI originality scores, though even after taking these other measures into account, a moderately strong association remains. Taken together, this suggests that FIQ is predicting something beyond what Torrance Suppose and Torrance Product can predict alone. The question then becomes, what are different measures of divergent thinking, specifically FIQ, measuring differently?

The fact that the FIQ stimuli provide prompts to idea search that are *both* ambiguous or open-ended [29–32, 41] *and* visual-spatial rather than exclusively verbal [66] is likely an important element in answering this question–as suggested by recent examinations of human and computational re-representation with stimuli such as the AUT and Pattern Meanings Test [25, 26]. Additionally, despite the surface similarity between the Design Product Ideation task and the Torrance Product task there are a number of notable differences. In the Torrance product improvement task, participants are provided a detailed verbal description of a single toy (e.g., a toy elephant) with an accompanying visual depiction and/or an actual physical toy, and the participant’s task is to think of all the ways to make the described toy more fun to play with. The product improvement task is thus quite closely focused on a single existing concrete object, with a specifically stated goal (adapting an existing toy product to make the toy more fun). In contrast, the Product Ideation Task describes a general domain of human activity (urban gardening, or picnics), and the participants are asked to generate a variety of different creative product ideas that could enhance a users’ engagement in those activities. The generated ideas could, in some instances, involve the aim of increasing the users’ fun or enjoyment, but may also involve many other viable objectives, such as enhancing the efficiency or ease of engaging in the activity, addressing obstacles to the activity (e.g., insects, wind, rain), promoting sustainability, considerations relating to aesthetics or fashion or social inclusiveness, accommodations for children or older adults or individuals with physical challenges, etc. The Product Ideation Task thus does not begin with an existing concrete product, requires a participant to imaginatively think of the designated activity domain from a variety of different viewpoints, with different possible goals, and user-needs in mind.

Future research should aim to directly measure the underlying cognitive functions involved in the FIQ and each of the divergent thinking measures used in this study, as well as the less-often used abstract Pattern Meaning Test stimuli of Wallach and Kogan (thus termed "WaKo" stimuli by [26]). Such research might, for example, implement a think-aloud procedure during the tasks, as was done for the AUT by Gilhooly et al. [24] and earlier for art, writing, and different problem-solving tasks [67, 68], or contrast across-item cumulative response times, as recently implemented for a comparison of the AUT and Consequences task [69]. With this information, it would be possible to use measures of divergent thinking more closely related to the target outcome as predictors of creative performance. For example, if the target outcome relies on mental rotation, verbal skills, and part-whole relations, a researcher could choose the divergent thinking tasks that most closely measure those processes.

An additional consideration for future research, that could help to measure and disentangle the underlying cognitive functions involved in each of the four divergent thinking measures, would be to improve upon the present scale for Perceptual Curiosity. The current scale from Litman and Spielberger [61] contains many fewer items assessing Perceptual Curiosity as compared to Epistemic Curiosity. Many of the Perceptual Curiosity items also overlap conceptually with Epistemic Curiosity, which is concerned with gaining new intellectual or conceptual knowledge rather than sensory stimulation. For example, one Perceptual Curiosity item asks about the respondent’s desire to visit art galleries or art museums, which would certainly involve visual sensation seeking, but may also be related to the intellectual stimulation involved in Epistemic Curiosity. A future measure of curiosity should address this concern by adding more items to the Perceptual Curiosity scale that are more specific to the construct. Epistemic Curiosity, too, demonstrated little predictive value for participants’ originality of Design Product Ideation. This contrasts with the outcome for the conceptually-related measure of Openness to experience, which, as noted above, was correlated with novel idea generation in Design Product Ideation at both pre-test and post-test and was also positively correlated with the individual divergent thinking measures both at pre-test (correlations between .20 and .36) and post-test (correlations between .27 and .42). In other research using the Epistemic Curiosity measure (Koutstaal, Kedrick, & Gonzalez-Brito, under review), this measure of dispositional or trait curiosity correlated especially strongly with measures of everyday creative activities and achievements. A similar outcome is observed in the current study, where Epistemic Curiosity is robustly associated with reported engagement in creative activities (*r* = .50, *p* < .001) and also unrealized creative ideas (*r* = .40, *p* < .001), but shows essentially no correlation with DPI originality. Based on findings that Openness to experience and prior creative training were significant in predicting DPI originality scores, future research should also evaluate what types of interventions are most effective at increasing Openness to experience or characteristics associated with Openness, as well as which specific forms of instruction are most effective at increasing novel divergent search–and creative perspective-taking–in "real world" tasks such as how to make urban gardening or outdoor picnics more effective, enjoyable, inclusive, sustainable, etc.

Contrary to our expectations, cognitive ability as assessed by the Shipley-2 measures of Abstraction and Vocabulary, also was not a significant predictor of originality on the Design Product Ideation task. Although the precise magnitude and nature of the contribution of general mental ability to creative thinking, and particularly divergent thinking, continues to be debated [54, 55, 70], more substantial correlations may be observed when using a broader set of indicators than the brief standardized assessments adopted here. Additionally, it might be noted that, although there were only slight and nonsignificant correlations between the measures of Abstraction and Vocabulary and originality on the DPI task, there were some moderate correlations between originality on the individual divergent thinking tasks and Abstraction (e.g., Abstraction-preSuppose *r* = .27, *p* = .007, Abstraction-preProduct *r* = .24, *p* = .017), and also between originality on the individual divergent thinking tasks and Vocabulary (with postProduct, *r* = .21, *p* = .04, with postAUT, *r* = .29, *p* = .005). The magnitude of these correlations with individual divergent thinking measures is not dissimilar to those recently reported in a meta-analytic update [56] across 67 studies and 467 coefficients on the relation between fluid intelligence and divergent thinking (average *r* = .23, 95% CI [.18, .28]) and across 28 studies and 137 coefficients for crystallized intelligence and divergent thinking (average *r* = .28, 95% CI [.22, .33]).

A consideration of the methods and participants of the current study introduces both potential strengths and possible limitations. In the current study we used what has been termed a "traditional" approach to the scoring of creativity [71], in which we asked trained novice raters to assess the fluency, flexibility, and originality of participants’ responses. There is ongoing evaluation of the advantages and disadvantages or downsides of different approaches to evaluating both creativity generally, and originality specifically [72–75]. Different methods have both merits and demerits. For instance, although some researchers have proposed that originality should be assessed by the relative statistical infrequency of responses, this method introduces its own problems. It emphasizes only the infrequency of responses as an aspect of originality–rather than a more nuanced and encompassing consideration of other contributors to originality such as playfulness or the many ways that an often-proposed idea nonetheless might be developed in an especially insightful, ingenious, or synergistic manner (e.g., a particularly innovative way in which a picnic blanket might be made to be self-cleaning). Frequency scoring also encounters challenges related to the comparability of scores across different samples (e.g., across samples with differing levels of expertise). Further, despite the appearance of greater objectivity, frequency scoring still requires item-by-item decisions regarding whether any given response is "similar enough" to a common response to be coded as such or, instead, is sufficiently different that it should be considered unique [75].

Although rater-based scoring also has drawbacks, it might be noted that, in their examination of different scoring approaches to the AUT, which is among the most widely administered assessments of divergent thinking, Vartanian et al. [71] reported significant positive correlations between rater-based scoring of originality and alternative methods including "snapshot" originality (*r* = .70). Other researchers have likewise shown that there can be very high correlations between the response ratings provided by a smaller set of trained raters and larger samples of raters (*r* = .83) for the AUT [72]. The strong-to-excellent interrater reliability that we observed for the various tasks and dimensions might, in part, be attributed to factors such as explicitly encouraging raters to take sufficient time to provide their evaluations, to take regular breaks, and emphasizing openness and receptivity to multiple perspectives–all factors that have been found to increase the reliability of the traditional scoring approach [72] and that were incorporated into the training of the raters in the current study.

Another consideration concerns the number of items that were included in each of the divergent thinking tasks and the key outcome measure of Design Product Ideation. For the key outcome measure, to reduce participant burden and maximize engagement, only one prompt (either urban gardening or picnics) was presented on each of the two testing occasions and presented for 10 minutes, thereby allowing sufficient time for reflection, incubation, and search from multiple perspectives. Similarly, for the AUT, Torrance Product, and Torrance Suppose only one item was provided on each testing occasion with participants given 5 minutes for each of these tasks. The FIQ, in contrast, involved the presentation of four items on each testing occasion. However, it is notable that each FIQ item was presented for only 40 seconds, and thus the total presentation time for all four items (under three minutes) was less than that for any of the other divergent thinking tasks, yet the FIQ consistently proved to be a significant incremental predictor of Design Product Ideation. This suggests that, in addition to providing desirable features such as being highly perceptual and sufficiently open-ended or ambiguous, the FIQ may offer an especially efficient measure of divergent thinking–and so could be more readily incorporated to supplement exclusive reliance on the AUT in time-constrained research protocols, such as those involving neuroimaging.

Amongst the strengths of this study are the conceptual replication it provides, and the comparatively large number of participants, including participants who sometimes might be excluded from similar studies, such as individuals who do not self-report normal or corrected-to-normal vision or are not native speakers of English. An additional factor, that is simultaneously a strength and a limitation, is that by the very fact of taking a creative design course, participants were likely to be more motivated and engaged in the creative assessment tasks compared to individuals from a more general population. A related point is the considerable prior creative experience of the participants. All reported at least 3 formal creative training experiences, such as in a class, and reported at least 14 informal creative training experiences, such as through work experience. While this factor likewise may have helped in promoting engagement in the creativity-related assessments, it could also act as a limiting factor in generalizing the results to a wider population.

In conclusion, as hypothesized, it was found that Design Product Ideation at both pre-test and post-test was predicted by originality of performance on one or more divergent thinking measures, but conjointly predicted by one or more personality, prior experience, or task-specific factors. Specifically, originality on the FIQ was consistently predictive on both the pre- and post-Design Product Ideation tasks; originality on Torrance Suppose at pre-test and Torrance Product at post-test were also predictive, whereas AUT originality was predictive only at pre-test. Additionally, the task-specific factor of perceived challenge of the DPI task, and the personality characteristic of Openness to experience, were predictive at pre-test and post-test respectively. We also found that originality on the perceptually-prompted ambiguous shapes FIQ task positively correlated with originality of Design Product Ideation, even after controlling for participants’ scores on other measures that correlated with Design Product Ideation.

Theoretically, our findings are in line with the componential or multi-factor theory of creativity [4–8]. From a methodological perspective, our findings point to the incremental predictive value of performance-based assessments of divergent thinking and argue against using a single measure of divergent ideation. Finally, from a practical standpoint, the findings reported here point to the value of both providing creativity-related training and of developing and sustaining contexts that promote the forms of exploration, experimentation, and risk-taking associated with Openness to experience.
